# Sequential algorithm for life threatening cardiac pathologies detection based on mean signal strength and EMD functions

**DOI:** 10.1186/1475-925X-9-43

**Published:** 2010-09-04

**Authors:** Emran M Abu Anas, Soo Y Lee, Md K Hasan

**Affiliations:** 1 Department of Electrical and Electronic Engineering, Bangladesh University of Engineering and Technology, Dhaka-1000, Bangladesh; 2 Department of Biomedical Engineering, Kyung Hee University, Kyungki 446-701, Korea

## Abstract

**Background:**

Ventricular tachycardia (VT) and ventricular fibrillation (VF) are the most serious cardiac arrhythmias that require quick and accurate detection to save lives. Automated external defibrillators (AEDs) have been developed to recognize these severe cardiac arrhythmias using complex algorithms inside it and determine if an electric shock should in fact be delivered to reset the cardiac rhythm and restore spontaneous circulation. Improving AED safety and efficacy by devising new algorithms which can more accurately distinguish shockable from non-shockable rhythms is a requirement of the present-day because of their uses in public places.

**Method:**

In this paper, we propose a sequential detection algorithm to separate these severe cardiac pathologies from other arrhythmias based on the mean absolute value of the signal, certain low-order intrinsic mode functions (IMFs) of the Empirical Mode Decomposition (EMD) analysis of the signal and a heart rate determination technique. First, we propose a direct waveform quantification based approach to separate VT plus VF from other arrhythmias. The quantification of the electrocardiographic waveforms is made by calculating the mean absolute value of the signal, called the mean signal strength. Then we use the IMFs, which have higher degree of similarity with the VF in comparison to VT, to separate VF from VTVF signals. At the last stage, a simple rate determination technique is used to calculate the heart rate of VT signals and the amplitude of the VF signals is measured to separate the coarse VF from VF. After these three stages of sequential detection procedure, we recognize the two components of shockable rhythms separately.

**Results:**

The efficacy of the proposed algorithm has been verified and compared with other existing algorithms, e.g., HILB [1], PSR [2], SPEC [3], TCI [4], Count [5], using the MIT-BIH Arrhythmia Database, Creighton University Ventricular Tachyarrhythmia Database and MIT-BIH Malignant Ventricular Arrhythmia Database. Four quality parameters (e.g., sensitivity, specificity, positive predictivity, and accuracy) were calculated to ascertain the quality of the proposed and other comparing algorithms. Comparative results have been presented on the identification of VTVF, VF and shockable rhythms (VF + VT above 180 bpm).

**Conclusions:**

The results show significantly improved performance of the proposed EMD-based novel method as compared to other reported techniques in detecting the life threatening cardiac arrhythmias from a set of large databases.

## Background

Ventricular Fibrillation (VF) and Ventricular Tachycardia (VT) are life-threatening cardiac arrhythmias generally observed in adults with coronary artery disease. In 1979, automatic external defibrillators (AEDs) were introduced to accurately analyze the cardiac rhythms and, if appropriate, advise/deliver a high-energy shock to those patients who suffer from coarse VF and VT of a rate above 180 bpm, combinedly known as the shockable rhythms [6]. Though a significant number of works have been published on this topic, the scope for development of more accurate and reliable techniques relaxing assumptions of certain previous works and incorporating features from diverse nature of the cardiographic signals is yet open. Based on separation capability, the algorithms available in the literature can be classified into categories such as, separating VF from VT [4,7,8], VF from normal sinus rhythm (NSR) [9], VF plus VT from nonVTVF [10], shockable rhythms from other ECG pathologies [5,11,12], VF from nonVF [1-4,13-24]. Comprehensively, the last two categories [25] are the most realistic for fruitful hospital management of cardiac abnormalities.

To separate VF from VT many efforts have been aimed at characterizing these abnormalities by means of diverse techniques such as the sequential hypothesis algorithm proposed by Thakor et al. [4], continuous wavelet transform [7], paired unipolar electrograms [8] etc. But only separating VF from VT is not useful for cardiac management. Because, in real life problems, other types of abnormalities are also present. A recent work is presented in [9] using the EMD technique to separate VF from NSR which shows almost 100% accuracy. But, when other types of pathology except the NSR and VF are present, poor accuracy is obtained. To separate VT plus VF from other arrhythmias, a time domain based complexity measure algorithm has been proposed in [10]. But it fails to show good performance due to its weakness in selecting a proper threshold value. Another approach has been reported in [5] to classify arrhythmias into two types: shockable and non-shockable signals. This work shows quite good accuracy but improvement area is still open. Various algorithms have been developed for classifying the abnormalities according to the last category. To separate VF from other arrhythmias, different methods were proposed based on different techniques of signal processing, such as the threshold crossing interval (TCI) algorithm [4], auto-correlation function (ACF) [13], probability density function method [14], VF-filter method [15], [16], [17], rate and irregularity analysis [18], [19], sequential hypothesis testing algorithm [20], [21], correlation waveform analysis [22], spectral analysis [3] and four fast template matching algorithms [23]. But these algorithms fail to show good performance when tested on a large database due to the some shortcomings in their reported algorithms. For example, the TCI method, based on a time domain technique, fails to detect the normal sinus rhythm (NSR) signal due to several factors, e.g., choice of 1-s analysis window, improper threshold etc. [24]. An improved version of this algorithm called the threshold crossing sample count (TCSC) method has been reported in [24] by removing some of the drawbacks of the TCI method. But the TCSC algorithm does not consider the shape of the ECG signal, therefore, it fails to classify VT into the nonVF group. On the other hand, the ACF relies on the regularity in NSR and irregularity in VF rhythms [26]. But practically, in most cases, there is no strict regularity found in the NSR signal and, therefore, the detection accuracy of the NSR signal by this method severely falls. The spectral analysis method successfully detects the nonVF signal from ECG arrhythmias. But in the detection of VF, this method shows poor accuracy due to the false detection of the VF signal with low peak frequency in the spectrum [26]. On the other hand, the Hilbert transform (HILB) [1] and phase space reconstruction (PSR) [2] algorithms employing phase space plot of the ECG signal demonstrate improved performance of VF detection. Because the phase space plot is based on the histogram of a signal, it does not consider the shape of this signal. Thus, to separate VT from VF when other arrhythmias are also present, these two methods are not very suitable.

In this paper, we propose a sequential detection algorithm based on the mean absolute strength and certain low-order intrinsic mode functions (IMFs) of the EMD analysis of the signal along with a simple rate determination technique. In our proposed algorithm, we not only separate VF but also VT from other arrhythmias. VT plus VF (VTVF) is separated from other arrhythmias in the first stage using an index called the mean absolute value (MAV). Then we decompose the VTVF signal into IMFs using the EMD technique to discriminate VF from VT. EMD was introduced in [27] for processing signals from nonlinear and non-stationary processes. Here, we apply the EMD technique to biomedical signals and particularly for ECG analysis. Next, a simple rate determination algorithm is utilized to classify VT according to the heart rate and to separate coarse VF from fine VF, amplitude of the VF signals are measured. Finally, this sequential ECG arrhythmias classification approach is interpreted as three different detection schemes, such as, VTVF from nonVTVF; VF from nonVF; shockable from non-shockable rhythms. While proposing an algorithm for detecting the shockable rhythms special care must be taken to make the specificity high. It will then ensure the false alarm generation probability of the AEDs low. But an algorithm with high specificity generally results in low sensitivity. To mitigate this contradictory requirement, detection of the shockable rhythms using a sequential algorithm is found to be more effective. At last, in the 'Results' Section, we compare our algorithm with different well-known algorithms available in the literature.

## Methods

### ECG signals

We use the MIT-BIH Arrhythmia Database (MITDB) [28], Creighton University Ventricular Tachyarrhythmia Database (CUDB) [29] and MIT-BIH Malignant Ventricular Arrhythmia Database (VFDB) [30] to evaluate our algorithm. The MITDB contains 48 files, 2 channels per file, each channel 1805 seconds long. The CUDB contains 35 files, 1 channel per file, each channel 508 seconds long. The VFDB contains 22 files, 2 channel per file, each channel 2100 seconds long. In our analysis, we choose episodes of 8-s long from the whole MIT-BIH arrhythmia and CU databases. We perform a continuous analysis by taking the data in steps of 1 sec. Thus, the total number of 8-s episodes collected from the MITDB and CUDB are (1805-7) × 48 × 2 = 172608 and (508-7) × 35 = 17535, respectively. Since, the VFDB includes ECG recordings of subjects who have experienced episodes of sustained VT and VF, we use this database for VF and VT episodes. By taking the ECG signal in steps of 1 sec we choose 4000 episodes of VF and 4000 episodes of VT from this database. Therefore, a total of 172608 + 17535 + 4000 + 4000 = 198143 episodes are used to compare our algorithm with other algorithms. Amongst these 198143 episodes, we have noticed some episodes which are annoted as the noise signals. Since, in this work we have no interest in these noise signals, we have omitted these noise episodes. Also, analysis of the distinct mode asystole signal is not presented here. Therefore, this type of ECG signal is not included into our complete dataset.

The complete dataset includes the following types of ECG signals.

1. Normal beat

2. Left bundle branch block beat (LBBB)

3. Right bundle branch block beat (RBBB)

4. Atrial premature beat (APC)

5. Aberrated atrial premature beat

6. Nodal (junctional) premature beat

7. Supraventricular premature or ectopic beat

8. Premature ventricular contraction (PVC) beat

9. Fusion of ventricular and normal beat

10. Atrial escape beat

11. Nodal (junctional) escape beat

12. Paced beat

13. Fusion of paced and normal beat

14. Unclassifiable beat

15. Blocked APC

16. Ventricular tachycardia

17. Ventricular fibrillation

To determine the discriminating threshold and verify its effectiveness, the complete dataset is divided into two subsets: training and test datasets. The training dataset is used to determine the thresh-old value. To check the efficacy of the threshold value determined from the training dataset, the test dataset is used. Both the datasets include all types of above mentioned rhythms. The training dataset includes:

1. (1805 - 7) × 23 × 2 = 82708 episodes from MITDB (file no. 100-109, 111-119, 121-124).

2. 2000 episodes of VF and 2000 episodes of VT from VFDB.

On the other hand, the test dataset includes:

1. (1805 - 7) × 25 × 2 = 89900 episodes from MITDB (file no. 200-203, 205, 207-210, 212-215, 217, 219-223, 228, 230-234).

2. 2000 episodes of VF and 2000 episodes of VT from VFDB.

### Classification of the ECG signals according to the AHA recommendations

According to the AHA recommendations, all ECG abnormalities are classified into following categories [6]:

1. Shockable rhythms

• 'coarse VF': any VF signal with an amplitude of > 200 *μV*.

• 'VT-hi': rapid ventricular tachycardia with a rate of > 180 bpm.

2. Non-shockable rhythms

• 'NSR': normal sinus rhythm.

• 'N': other arrhythmia, including supraventricular tachycardia, sinus bradycardia, LBBB, RBBB, APC and PVC beats.

• 'Asyst': asystole; ECG signal with a peak-to-peak amplitude of < 100 *μV*, lasting more than 4 s.

3. Intermediate rhythms

• 'VT-lo': slow ventricular tachycardia with a rate of < 180 bpm.

• 'fine VF': any VF signal with an amplitude in the range 100 - 200 *μV*.

It is clear from this classification that VT is divided into two categories according to heart rate; 'VT-hi' and 'VT-lo'. This VT classification considers border heart rate as 180 bpm. It is, however, not strict. It may be in the range 150 - 180 bpm. AEDs only advise/deliver shock to shockable rhythms, and intermediate rhythms are treated in a different way called anti-tachycardia pacing.

### Detection of VTVF from other arrhythmias

**Figure 1:**
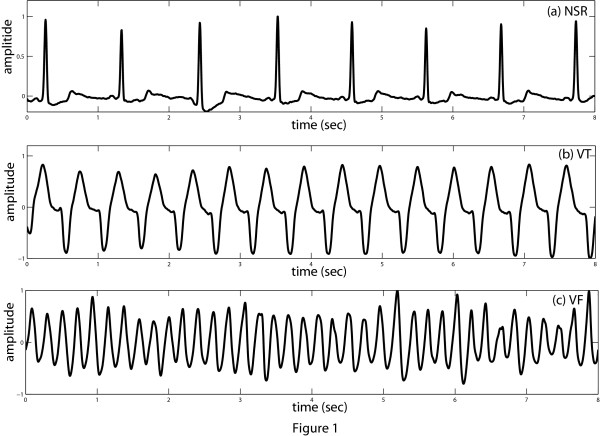
**Characteristics of NSR, VT, VF signals**. Characteristics of different variety of ECG signals (a) NSR episode, (b) VT episode, and (c) VF episode.

To detect the life threatening cardiac arrhythmias, VT and VF, from other arrhythmias, we propose to use a property that does not match with that of any nonVTVF signal. Typical ECG waveforms of NSR, VT and VF are given in Figure 1. Here, NSR is treated as the representative of nonVTVF signals. The three waveforms are plotted in the same scale. From this figure we see that the width of the QRS complex is different for different arrhythmias. For NSR, it is noticed that the QRS interval is normally 0.06 - 0.10 sec and in case of VT, the QRS complex is more wider (> 0.10 sec). In VF, no QRS complex is noticed. On the other hand, P waves are normal (upright and uniform) in the NSR waveform and in case of VT and VF signal, no P waves are observed [31].

The distinguishable morphological characteristics of these three groups, namely nonVTVF, VT and VF can be quantified using a term called the absolute strength of a signal. The absolute strength or the mean of absolute value (*MAV*) of a signal *x*(*n*) of length *N *is defined as

(1) $$MAV=\frac{1}{N}\sum_{n=0}^{N-1}\left|x\left(n\right)\right|$$

Here, *n *stands for the number of samples within the chosen length. In case of NSR, the main representative of the nonVTVF group, the duration of the QRS complex is small as compared to one ECG period as illustrated in Figure 1(a). It is also observed from this figure that the NSR signal level is low for most of the time in an ECG cycle. Therefore, the absolute signal level of the QRS complexes dominates in the summation of *MAV *calculation (eqn. (1)). A low *MAV *is thus obtained for such episodes. In case of VT, we see that the QRS complex is much wider than that of NSR, and the ECG signal hardly goes through the baseline as is the case for VF. Therefore, the *MAV *of VT and VF for a fixed duration window is comparatively larger than that for the NSR.

**Figure 2:**
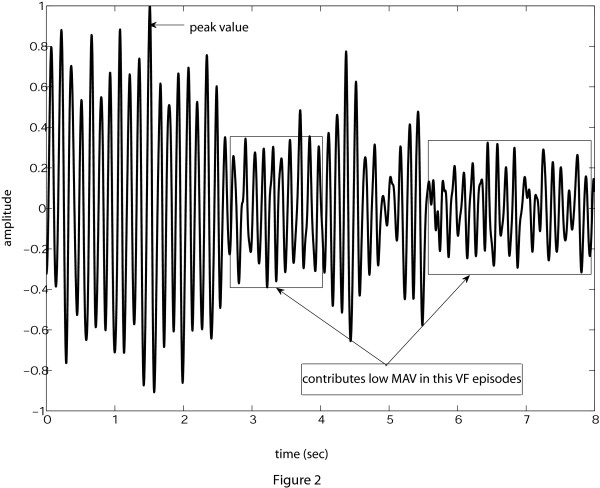
**Choice of analysis window**. Effect of choice of 2-s analysis window duration on *MAV*.

Before calculating the total *MAV *of a ECG signal, first it is necessary to normalize the ECG signal because the ECG signals collected from the different databases have different dynamic value. Another important thing to be noted is that, to use the *MAV *as the threshold parameter, we need to properly choose the analysis window duration. To understand the reason behind the necessity to appropriately choose the analysis window duration, consider a normalized VF episode of 8-s length from *cu*01*m *file of CU database shown in Figure 2. If we choose an 8-s episode length, then it may include a damped VF signal, as shown in figure 2, where most of the signal samples fall in the low amplitude range, and the MAV becomes low (e.g., 0.2577). Therefore, it is necessary to make the analysis window length small. Choosing an analysis window of too small duration (say, 1-s) creates the same problem as observed in the TCI method. Here, we choose the 2-s window for analysis. After calculating the *MAV *of this 2-s analysis window, we shift the analysis window by 1-s successively for other segments of 2-s within the 8-s ECG episode and calculate the *MAV *again. After completion of shifting the analysis window to cover the whole decision frame, we average all the *MAV *s found in each stage and finally *MAV *= 0.34 is found which is higher than that obtained for the 8-s analysis window. In this way, by appropriately selecting the analysis window length in calculating the *MAV *, we can overcome the effect of damped behavior of the ECG signal.

Observation of other nonVTVF ECG waveforms such as Premature Ventricular Contraction (PVC), Premature Atrial Contraction (PAC), Supraventricular Tachycardia (SVT) etc. reveals that these abnormalities also have low *MAV *compared to VT and VF. For example, PVC arrhythmia has small *MAV *because a PVC beat contains only wide QRS complex and no P waves or T waves are associated with this abnormal beat [31]. Thus, we can use *MAV *as the performance index to discriminate the VTVF from other arrhythmias.

In ECG analysis, it is important that we choose the episode length or decision frame appropriately. Decision frame should be taken in such a way that is neither too short to make a false alarm nor too long to cause severe cardiac arrest. Decreasing the episode length from its optimum value results in a low accuracy but quick detection. On the contrary, increasing the episode length improves the accuracy up to a certain level but requires longer detection time.

The whole process of separating VT plus VF from other arrhythmias can be described as in the following:

1. Choose a segment of ECG signal of *L_e_*-second duration. This segmented ECG signal of *L_e_*-second duration should be stored for the second stage.

2. The segment of the ECG signal is preprocessed using the well-known filtering process as used in [32], which is carried out in a MATLAB routine, called *filtering.m *[33]. The filtering algorithm works in four successive steps.

• First, the mean value is subtracted from the signal.

• Second, a moving average filter is applied in order to remove the power line noise.

• Third, a drift suppression is carried out by a high pass filter with a cut-off frequency of 1 Hz.

• In the last step, a low pass Butterworth filter with a cut-off frequency of 30 Hz is applied in order to suppress the high frequency noise like interspersions and muscle noise.

All filters in the preprocessing step is implemented using the Matlab routine 'filtfilt' function.

3. Then, choose a smaller segment *x*(*n*) from the ECG signal of *L_e_*-second duration in such a way that the length of the segment is 2-s. If the sampling frequency of the ECG signal is *F_s _*samples/s, then the total sample within this segment (*N*) is 2*F_s_*. For example, the sampling frequency of the ECG signal of the MITDB is 360 smaples/sec. Thus the length of the smaller segment *N *is 2 × 360 = 720 samples.

4. Next, divide the smaller segment *x*(*n*) by the maximum absolute value found in that segment.

5. Calculate the *MAV *using (1).

6. Shift the window by 1-s successively for other segments of 2-s within the *L_e_*-second ECG episode and go through step (4) to (5).

7. Make decision on every *L_e_*-second ECG episode (*L_e _*≥ 2) by averaging the *L_e _*- 1 consecutive values of *MAV *obtained from the *L_e _*- 1 consecutive 2-s segments with 1-s step. The average value, *MAV_a _*for an *L_e_*-second episode is calculated as

(2) $$MAV_{a}=\frac{1}{L_{e}-1}\sum_{i=1}^{L_{e}-\;1}MAV_{i}$$

where *MAV_i _*is the value of *MAV *in the *i*-th 2-s stage.

**Figure 3:**
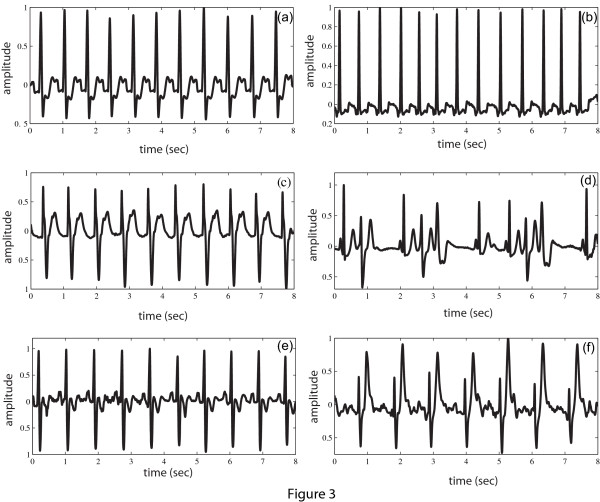
***MAV *of different ECG signals**. ECG waveform and the *MAV_a _*values of different nonVTVF pathologies. (a) Left bundle branch block beat, *MAV_a_*= 0.1649; (b) Nodal (junctional) premature beat, *MAV_a _*= 0.0954; (c) High rate supraventricular tachycardia, *MAV_a _*= 0.1372; (d) Premature ventricular contraction, *MAV_a _*= 0.1475; (e) Right bundle branch block beat, *MAV_a _*= 0.1571; (f) Paced beat, *MAV_a _*= 0.2166.

We calculate the *MAV_a _*of the three pathologies shown in Figure 1 and are obtained as 0.0765 (NSR), 0.3954 (VT) and 0.4116 (VF). To verify the effectiveness of the MAV index for separating the non-VTVF arrhythmias from the VTVF arrhythmias, other nonVTVF representatives namely, left bundle branch block beat, nodal (junctional) premature beat (rate ≈ 100 bpm), high rate supraventricular tachycardia (rate ≈ 100 bpm), premature ventricular contraction, right bundle branch block beat and paced beat are chosen from the ECG databases. These six pathologies are demonstrated in Figure 3 and their *MAV_a _*are 0.1649, 0.0954, 0.1372, 0.1475, 0.1571, 0.2166, respectively. Certainly, there is a clear separation of these *MAV_a _*values with those obtained from VT and VF episodes.

**Figure 4:**
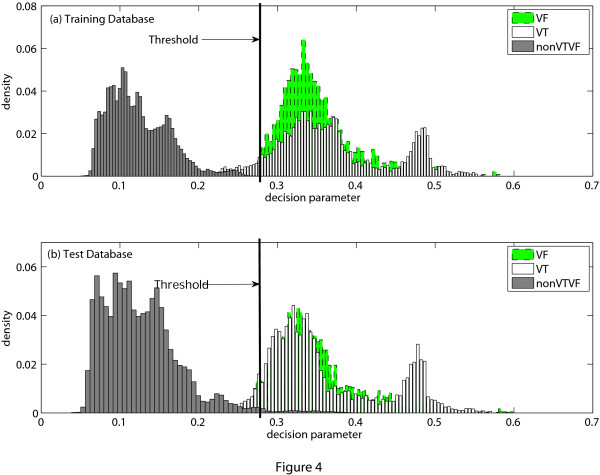
**Probability histogram of *MAV_a_***. Probability histogram of the decision parameter *MAV_a_*. (a) Training database, (b) Test database.

If *MAV_a _*is greater than a certain threshold *MAV_d_*, VTVF is detected. To determine the thresh-old value, training dataset is used. Figure 4 shows the probability distribution of *MAV_a _*of the training dataset and the test dataset. The threshold value is selected from the probability distributions of the training dataset shown in Figure 4(a) and we have chosen *MAV_d _*= 0.27 for *L_e _*= 8-s to ensure high specificity and also good sensitivity. It is also noticed from Figure 4(b) that when we apply this threshold to the test dataset, high accuracy is still obtained.

### Separation of VF from VTVF

Now that we have separated VTVF from other arrhythmias. In this stage, we separate VF from VT. Before we explain our motivation for using the EMD technique, we briefly describe what EMD is.

#### EMD Preliminaries

EMD is a signal decomposing method which is fully data-driven and does not require any *a priori *basis function [27,34]. The aim of the EMD is to decompose the signal into a sum of intrinsic mode functions (IMFs). An IMF is a function that satisfies two conditions: (1) in the whole data set, the number of extrema and the number of zero crossings must either be equal or differ at most by one; and (2) at any point, the mean value of the envelop defined by the local maxima and the envelop defined by the local minima is zero. An IMF represents the oscillatory mode embedded in the data as a counter-part to the simple harmonic function used in Fourier analysis [35].

Given a signal *x*(*n*), the starting point of the EMD is the identification of all the local maxima and minima. All the local maxima are then connected by a cubic spline [36] curve as the upper envelop *e_u_*(*n*). Similarly, all the local minima are connected by a spline curve as the lower envelop *e_l_*(*n*). The mean of the two envelops is denoted as *m*_1_(*n*) = [*e_u_*(*n*)+*e_l_*(*n*)]/2 and is subtracted from the signal. Thus the first component *h*_1_(*n*) is obtained as

(3) $$x(n)-m_{1}(n)=h_{1}(n)$$

The above procedure to extract the IMF is called the *sifting *process. Ideally, *h*_1_(*n*) should be an IMF, as the construction of *h*_1_(*n*) seems to have been made to satisfy all the requirements of IMF. Since *h*_1_(*n*) still contains multiple extrema in between zero crossings, the sifting process is performed again on *h*_1_(*n*). This process is applied repetitively to the proto- IMF *h_k_*(*n*) until the first IMF *c*_1_(*n*), which satisfies the IMF condition, is obtained. Couple of stopping criteria are used to terminate the sifting process [27]. A commonly used criterion is the value of standard deviation, SD, computed from the two consecutive sifting:

(4) $$\text{SD}=\sum_{n=0}^{N}\frac{|h_{1(k-1)}(n)-h_{1k}(n){|}^{2}}{h_{1(k-1)}^{2}(n)}$$

where, *N *is the total number of samples in *x*(*n*). When the SD is smaller than a threshold, the first IMF *c*_1_(*n*) is obtained. Then *c*_1_(*n*) is separated from the rest of the data by

(5) $$x(n)-c_{1}(n)=r_{1}(n)$$

It is to be noted that the residue *r*_1_(*n*) still contains some useful information. We can therefore treat the residue as a new signal and apply the same sifting process to obtain

(6) $$r_{i-1}(n)-c_{i}(n)=r_{i}(n),\;\;i=1,\;\ldots ,\;q$$

The whole procedure terminates when either the component *c_q_*(*n*) or the residue *r_q_*(*n*) becomes very small or when the residue *r_q_*(*n*) becomes a monotonic function. Combining (5) and (6) yields the EMD of the original signal,

(7) $$x(n)=\sum_{i=1}^{n}c_{i}(n)+r_{q}(n)$$

The results of the decomposition are *q -- *intrinsic modes and a residue. The lower order IMFs capture the fast oscillation modes while the higher order IMFs typically represent the slow oscillation modes present in the underlying signal [27,37]. An example illustrating the Empirical Mode Decomposition is given in the 'Appendix' section.

**Figure 5:**
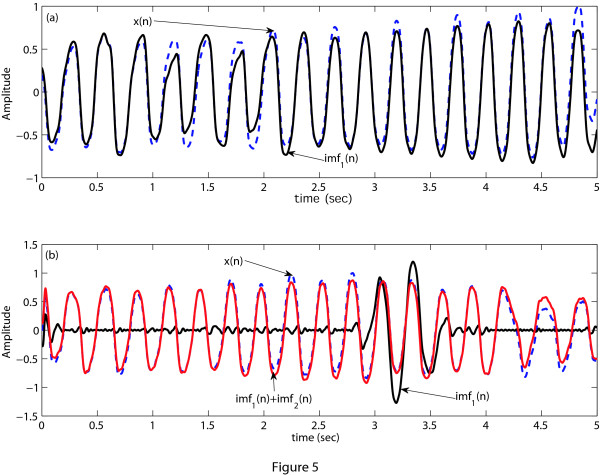
**EMD decomposition of VF**. EMD decomposition of a VF episode. (a) ECG signal *x*(*n*) and its first IMF of a VF episode without high frequency noise, (b) only the first IMF and the sum of the first two IMFs of a VF episode *x*(*n*) with high frequency noise.

**Figure 6:**
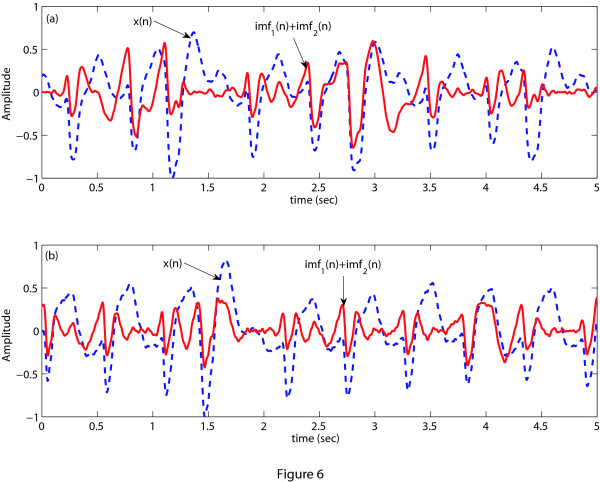
**EMD decomposition of VT**. ECG signal and the sum of the first two IMFs of a VT episode. (a) Noise free VT signal, (b) Noise corrupted VT signal.

As mentioned earlier, the VT waveform contains the QRS complex but it is absent in the VF wave-form. The asymmetry of the QRS complex with respect to the baseline gives rise to asymmetric signal envelopes which are comprised of local maxima and minima. Another interesting thing to be noted is that in case of VT, comparatively short duration of the QRS complex results in a wideband ECG signal. On the other hand, the QRS complex is absent in VF and as a result this pathology has more symmetric envelopes than do other abnormalities and thus possesses narrowband characteristics. Therefore, to separate VF from VT, the EMD technique can effectively use the factors of narrowband/wideband characteristics and symmetry/asymmetry property of a signal's envelopes. Now, we apply the EMD technique on a VF episode to decompose it into IMFs and plot the original ECG signal *x*(*n*) along with its first IMF as shown in Figure 5(a). From Figure 5(a) we can say that in case of VF, its first IMF is very much close to the original ECG signal. This is be-cause the VF has certain properties that well match the properties of the IMF as stated above. As the EMD technique cannot decompose an IMF signal further, therefore, in case of a VF episode, there is a unique relationship between the ECG signal and its first IMF. Here, unique relationship means that the original ECG signal and its first IMF is very much similar. In some cases high frequency noise still remains in the ECG signal after preprocessing. Therefore, when we apply EMD to decompose the VF signal, the first IMF captures this high frequency noise as the fast oscillation mode illustrated in Figure 5(b). To overcome this effect we consider the sum of the first two IMFs instead of using only the first one. We can observe from Figure 5(b) that unique relationship still exists between the ECG signal and the sum of first two IMFs for the VF episode. In case of VT, this unique relationship or similarity between the ECG signal and the sum of its first two IMFs does not hold as illustrated in Figure 6 for both noise free and noise corrupted VT signals.

To exploit the property of unique relationship between the ECG signal and the sum of its first two IMFs that exists in case of the VF only, sum of the first two IMFs from the ECG signal is subtracted and the *MAV *of the difference signal is calculated. Since, the dynamic range of the ECG signal varies from database to database, we normalize this *MAV *with respect to the original ECG signal. In case of a VF episode, the normalized *MAV *or *NMAV *of the difference signal is very small than that of a VT episode. Here, we choose a 2-s analysis window as in the previous case. But in this case, the performance index (*NMAV *) is less sensitive to the analysis window length.

The process of detecting VF from VTVF can then be described as below:

1. First, choose a segment *x*(*n*) of duration 2-s and *N *samples from the previously saved ECG signal of *L_e_*-second duration.

2. At this stage, the ECG signal is preprocessed in three successive steps.

• First, the mean value is subtracted from the signal.

• Second, a drift suppression is carried out by a high-pass filter with a cut-off frequency of 1 Hz.

• In the last step, a low-pass Butterworth filter with a cut-off frequency of 20 Hz and order 12 is applied to suppress the high frequency information.

3. Apply EMD on *x*(*n*) and determine

$$imf_{12}(n)\;=\;imf_{1}(n)+imf_{2}(n)$$

where, *imf*_1_(*n*) and *imf*_2_(*n*) denotes the first and second IMFs, respectively.

4. Then, calculate the difference between the original signal and sum of its first two IMFs,

$$e(n)=x(n)-imf_{12}(n)$$

5. The normalized *MAV *of *e*(*n*) used as the index for discriminating VF from VT is calculated as

$$NMAV=\frac{\frac{1}{N}\sum_{n=0}^{N-1}\left|e\left(n\right)\right|}{\frac{1}{N}\sum_{n=0}^{N=1}\left|x\left(n\right)\right|}$$

6. Shift the window by 1-s successively for other segments of 2-s within the *L_e_*-second ECG episode and go through step (ii) to (iv).

7. Make decision on every *L_e_*-second ECG episode (*L_e _*≥ 2) by averaging *L_e _*- 1 consecutive values of *NMAV *obtained from *L_e _*- 1 consecutive 2-s data segments with 1-s step. The average value *NMAV_a _*for an *L_e_*-second episode is calculated as

(8) $$NMAV_{a}=\frac{1}{L_{e}-1}\sum_{i=1}^{L_{e}-\;1}NMAV_{i}$$

where *NMAV_i _*is the value of *NMAV *in the *i*-th 2-s stage.

**Figure 7:**
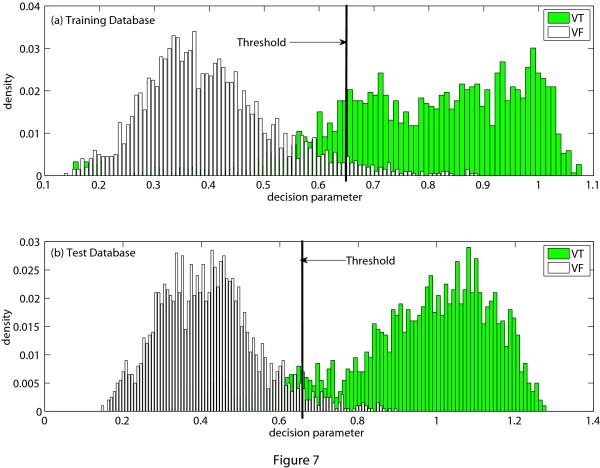
**Probability histogram of *NMAV*_*a*_**. Probability histogram of the decision parameter *NMAV_a_*. (a) Training database, (b) Test database.

Applying the above stated process, the *NMAV_a _*are obtained as 0.08 (for Figure 5(b)), 0.97 (for Figure 6(a)) and 0.93 (for Figure 6(b)). If *NMAV_a _*is less than a certain threshold *NMAV_d_*, VF is detected, otherwise VT is detected. The threshold value *NMAV_d _*is selected by a process as described before using the training dataset. As in this stage we separate VF from VT, therefore, the training and test datasets include only VF and VT episodes. This threshold value is then applied to the test dataset. Figure 7 shows the probability distribution of *NMAV_a _*of the training dataset and the test dataset. From the training dataset, we have chosen *NMAV_d _*= 0.65 for *L_e _*= 8-s to ensure that both VF and VT detection accuracies are good. It is also noticed from Figure 7(b) that the threshold value calculated from the training dataset can be applied to the test dataset maintaining almost the same accuracy as found from the training dataset.

### Classification of VT and VF according to the AHA recommendations

**Figure 8:**
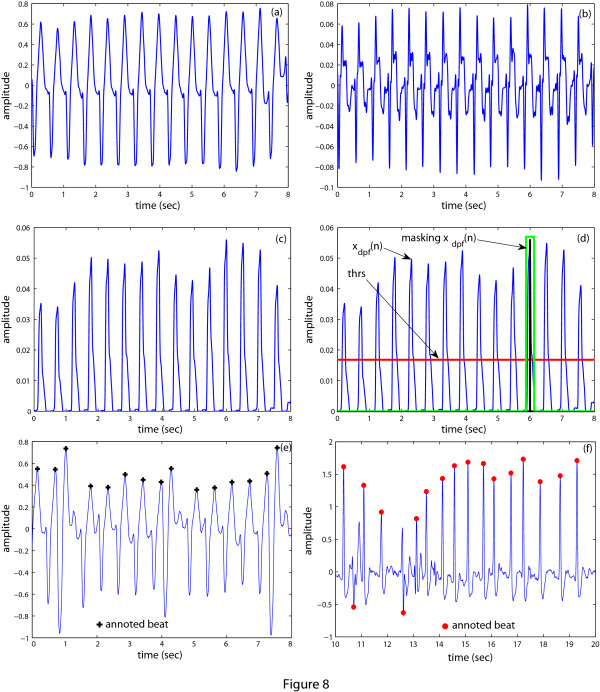
**Heart rate calculation**. ECG waveforms in different stages of the heart rate determination scheme (a) Preprocessed ECG signal, *x*(*n*); (b) First derivative, *x_d_*(*n*); (c) Filtered ECG signal, *x_dpf _*(*n*); (d) Determination of the threshold level and masking of *x_dpf _*(*n*). (e)-(f) Two episodes are taken to check the efficiency of the proposed heart rate determination technique and the results of the proposed technique matched with the annotations.

As only the certain classes of VTs and VFs require high-energy shock for treatment, it is necessary to classify the VT and VF according to the heart rate and amplitude, respectively. Since, the heart rate calculation is complicated than the amplitude determination, hence at first we propose a technique to determine the heart rate. The heart rate in bpm is defined as the number of QRS complexes that occur in 60 sec. To determine the heart rate of an ECG signal, first derivative of the ECG signal is utilized. The reason behind the choice of the first derivative of the ECG signal is to utilize the high slope of the QRS complex. Figs. 8(a) and 8(b) show the VT signal and its first derivative. Figure 8(b) illustrates that when QRS complexes occur, correspondingly there is a high value (both in positive and negative part) in the first derivative signal. We consider only the positive part of the first derivative signal. Then this signal is filtered to enhance the QRS complexes further. From this filtered signal shown in Figure 8(c), the heart rate is easily calculated. The whole process of determining the heart rate of the ECG signal is described below:

1. First, choose a segment *x*(*n*) of duration *L_e_*-second and *N *samples from the previously saved ECG signal and then perform preprocessing as stated in Section.

2. Calculate the first derivative (*x_d_*(*n*)) of *x*(*n*).

$$x_{d}(n)=x(n)-x(n-1)$$

The waveform of *x_d_*(*n*) is shown in Figure 8(b).

3. Keep only the positive part of *x_d_*(*n*).

$$x_{dp}(n)=\{\begin{array}{cc} \begin{array}{r} x_{d}(n)\\ 0 \end{array} & \begin{array}{l} if\;x_{d}(n)\geq 0\\ ;otherwise \end{array} \end{array}$$

4. Apply the moving average filter on *x_dp_*(*n*) and find *x_dpf _*(*n*).

$$x_{dpf}(n)=\sum_{k=0}^{k=\alpha }x(n\;-k)$$

where, *α *= *F_s_*/10 and *F_s _*is the sampling frequency. If *α *is not an integer, then it is rounded to the nearest integer value. The waveform of *x_dpf _*(*n*) is shown in Figure 8(c).

5. Determine the maximum value (*C*) and the corresponding peak index (*I*) of *x_dpf _*(*n*) and calculate the threshold value (*T_h_*) from *C*.

$$\begin{array}{lll} C & = & \max\{x_{dpf}(n)\}\\ T_{h} & = & C\times \beta \end{array}$$

where, *β *is a properly chosen constant. Here, we choose *β *= 0.25.

6. Store the peak index (*I*) and mask *x_dpf _*(*n*) around this position.

$$x_{dpf}\;(I-\gamma :I+\gamma )\;=\;0$$

where, γ = *F_s_*/8; if γ is not an integer, then it is rounded to the nearest integer value.

7. Now, calculate again the maximum value (C) of *x_dpf_*(*n*) and go through step (vi) until C goes below the *T_h_*.

8. Determine the total number of peaks (*N_p_*) those are above *T_h _*and calculate *H_R_*.

$$H_{R}=\frac{N_{p}\times 60}{L_{e}}bpm$$

If the heart rate of the VT signal is greater than 180 *bpm*, then this VT is called the shockable VT. As the decision of shockable or intermediate VT is dependent on the heart rate of the episode, hence, we calculate the total number of QRS beats in a episode. Now, to check the efficiency of the heart rate determination algorithm, two episodes selected are shown in Figs. 8(e)-(f). At first, the total number of QRS beats in these episodes are determined from the annotation. Then, the proposed derivative based heart rate determination algorithm is used to calculate the total number of QRS beats and it is found to be 15 beats for Figure 8(e) and 17 beats for Figure 8(f). In both cases, the total number of QRS complexes obtained by using our algorithm are the same as determined from the annotation. Thus, this heart rate determination method, though simple, may be used to calculate the heart rate of an ECG episode. However, in more complicated cases any standard heart rate determination algorithm re-ported in the literature [38,39] may be adopted to classify the VT. On the other hand, the amplitude of the VF signal is determined by taking the maximum value of the absolute VF signal within a episode. If the amplitude is greater than 200 *μV*, than this VF is called the coarse VF.

### Quality Parameters

The quality parameters, we have used for the assessment of algorithms, are sensitivity, speci city, positive predictivity, and accuracy. For 'VTVF' detection, the first four parameters are defined by

$$Sensitivity=\frac{No.\;of\;detected\;“VTVF”}{No.\;of\;true\;“VTVF”}$$

$$Specificity=\frac{No.\;of\;detected\text{​}\;“nonVTVF”}{No.\;of\;true\;“nonVTVF”}$$

$$\begin{array}{l} Positive\;Predictivity\;(\;Pos.Pred.)\;=\\ \frac{No.\;of\;detected\;“VTVF”}{No.\;of\;cases\;classified\;by\;algorithm\;as\;“VTVF”} \end{array}$$

$$Accuracy\;=\frac{No.\;of\;true\;decisions}{No.\;of\;all\;decisions}$$

For 'VF' and 'shockable rhythm' detection, the definition of these four quality parameters contain 'VF' and 'shockable rhythm' in place of 'VTVF', respectively. While calculating these four quality parameters to judge the effectiveness of an algorithm, in case of any unsatisfactory results obtained, the values of the respective thresholds were adjusted in order to obtain the best possible results.

## Results and Discussion

**Figure 9:**
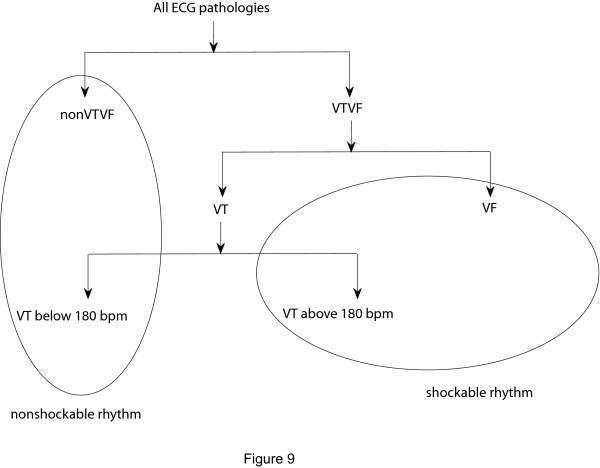
**Classification of different ECG signals**. Classification of different types of ECG pathologies.

The full classification of different ECG pathologies is shown in Figure 9. To compare our algorithm with other reported algorithms in the literature, our classification approach can be interpreted as three different ECG arrhythmias identification schemes; such as

1. VTVF and nonVTVF

2. VF and nonVF (nonVTVF+VT)

3. shockable (VF+VT above 180 bpm) and non-shockable (nonVTVF+VT below 180 bpm)

Since the annotated files do not contain enough low-amplitude signals (fine VF), therefore, this type of signal is not addressed in this identification scheme and the VF signals in the shockable rhythms are actually coarse VF. Now, this section is divided into three subsections and each subsection presents the results of each identification scheme.

### Detection of VTVF from other arrhythmias

First, we test the separability of our algorithm between the two classes of ECG signals, i.e., 'VTVF' and 'nonVTVF' against the annotated decisions suggested by the cardiologists in the respective databases. We compare our algorithm with the complexity measure algorithm [10] and the results are shown in Table 1. Comparative results illustrate that our algorithm shows better performance than the complexity measure algorithm. Also notice that the accuracy of the proposed MAV scheme is significantly higher than that of the complexity measure algorithm. Thus our simple and fast algorithm can separate VTVF from nonVTVF with higher specificity and sensitivity simultaneously. In this case, we had to change the threshold value of the CPLX algorithm from that defined in [10] to obtain higher sensitivity.

**Table 1:** Quality parameters of VTVF detection.

Algorithm	Quality Parameters for VTVF detection			
	
	Sensitivity (%)	Specificity (%)	Pos. Pred. (%)	Accuracy (%)
MAV	93.69	99.39	89.46	99.07

CPLX [10]	48.95	79.48	11.82	77.86

### Detection of VF from other arrhythmias

VF occurs at the clinically crucial stage of human being. As mentioned earlier, while detecting VF from the other arrhythmias in the first stage, we should make the specificity high because a low specificity may risk patient's life by generating a false alarm to provide a high energy shock as treatment to save his/her life. But our proposed sequential algorithm leads to a high specificity. Quality parameters of our proposed algorithm with some other well-known algorithms are shown in Table 2. It is clear that our algorithm shows higher sensitivity compared to all other algorithms with very good specificity (99.32%). To obtain higher specificity, we had to change the critical threshold parameter of the HILB and PSR methods from that defined in the respective papers. To compare different methods independent of the value of the decision thresholds, the critical threshold parameter in the decision stage of the algorithm is varied. By varying the threshold, we can vary specificity and sensitivity as shown in Table 3. This table illustrates that our proposed method performs much better than the other VF detection algorithms.

**Table 2:** Quality parameters of different VF detection algorithms for *L*_*e *_= 8-s.

Algorithm	Quality Parameters for VF detection			
	
	Sensitivity (%)	Specificity (%)	Pos. Pred. (%)	Accuracy (%)
TCI	94.64	65.08	8.46	66.05

SPEC	41.42	99.57	76.67	97.65

HILB	71.76	98.87	68.41	97.98

PSR	63.69	99.05	69.57	97.88

TCSC	80.19	98.53	65.66	97.96

MAV & EMD	86.49	99.32	81.27	98.90

**Table 3:** Performance comparison of different algorithms for a fixed specificity and for *L*_*e *_= 8-s.

Algorithm	Sensitivity if Specificity =		
	
	99%	98%	96%
TCI	0.33	0.73	5.73

SPEC	65.2	69.35	74.93

HILB	65.32	84.79	91.58

PSR	62.17	77.53	92.40

TCSC	65.07	84.23	93.94

MAV & EMD	89.32	94.76	95.61

### Detection of shockable rhythms from other arrhythmias

This subsection presents the results of our last identification scheme which classifies the ECG pathologies into two groups: shockable and non-shockable rhythms. To compare our algorithm with the reported algorithm in [5], some modifications in the threshold values are made to accommodate unequal episode lengths (*L_e_*). Our proposed algorithm considers *L_e _*= 8-s where *L_e _*= 10-s was considered in [5]. Modifications are shown in Table 4. For example, if *Count*1 < 250 for *L_e _*= 10-s, then for *L_e _*= 8-s *Count*1 < 250 10 * 8 or *Count*1 < 200.

**Table 4:** Modifications in the threshold values proposed in [5] (*C*1 = *Count*1, *C*2 = *Count*2, *C*3 =*Count*3).

**Condition No**.	for *L_e _*= 10-s	for *L_e _*= 8-s
1	*C*1 < 250, *C*2 > 950 and *C*1 × *C*2/*C*3 < 210	*C*1 < 200, *C*2 > 760 and *C*1 × *C*2/*C*3 < 168

2	250 ≤ *C*1 < 400, *C*2 < 600 and *C*1 × *C*2/*C*3 < 210	200 ≤ *C*1 < 320, *C*2 < 480 and *C*1 × *C*2/*C*3 < 168

3	*C*1 ≥ 250 &*C*2 > 950	*C*1 ≥ 200 &*C*2 > 760

4	*C*2 ≥ 1100	*C*2 ≥ 880

**Figure 10:**
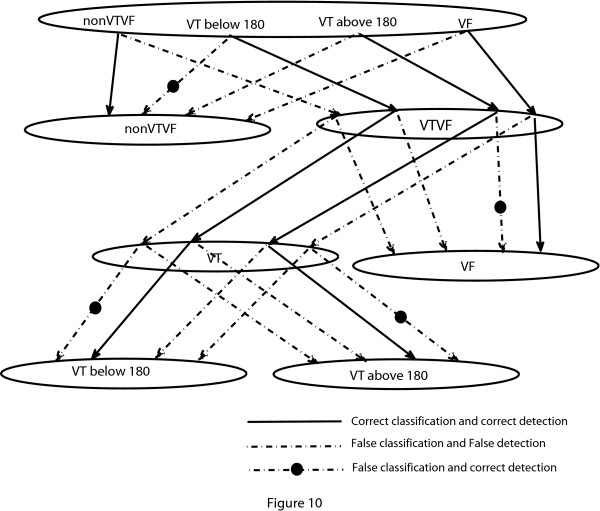
**Effect of false classification**. Effect of false classification on the detection of shockable rhythms

Here, as we concentrate only on the shockable and non-shockable rhythms, some classification errors may not result in detection errors. For example, from Figure 10 we see that a classification error occurs when a VT above 180 bpm is falsely detected as VF in the second stage. Since a VT above 180 bpm (one type of shockable rhythms) is falsely mapped into the VF group, which is also in the class of shockable rhythms, therefore, this classification error does not make any detection error as long as shockable rhythm is our concern. Using the modified threshold values mentioned in Table 4, the results obtained are presented in Table 5. As can be seen, our algorithm performs better than the count [5] algorithm in every index in detecting the shockable rhythms correctly.

**Table 5:** Quality parameters for the detection of shockable rhythms using *L*_*e *_= 8-s.

Algorithm	Quality parameters for the detection of shockable rhythms			
	
	Sensitivity (%)	Specificity (%)	Pos. Pred. (%)	Accuracy (%)
MAV & EMD	91.09	99.42	90.71	99.21

Count [5]	88.90	99.29	85.99	98.93

## Conclusions

A novel method for the identification of life threatening cardiac abnormalities from other arrhythmias has been presented. Performing sequential signal processing, we have detected these cardiac abnormalities with good accuracy. It has been shown that the proposed algorithm based on the MAV parameter and EMD technique can detect the VT plus VF signals correctly from other arrhythmias, and the accuracy level remains higher than that of other reported techniques. The effectiveness of the pro-posed technique has been demonstrated using standard databases over a vast range of both normal and abnormal ECG records. The *MAV *index successfully separates the VTVF arrhythmias from different types of abnormalities. And the other parameter *NMAV *which is calculated using the IMFs of the EMD technique can successfully separate VF from VTVF. Finally, a fast and simple heart rate determination technique is used to separate the high rate VT. Consistent results have been obtained by applying our algorithm on different well-known databases namely, MIT-BIH database, CU database and MIT-BIH Malignant Ventricular Arrhythmia database. Determination of the threshold parameters from the training dataset and then their successful application on the test dataset proves that the proposed parameters are universal. Some signal episodes were very difficult for classification even by expert cardiologists. Accuracy of our proposed technique slightly falls due to these confusing episodes. The algorithm presented here has strong potential to be applied in clinical applications for accurate detection of life threatening cardiac arrhythmias.

## Competing interests

The authors declare that they have no competing interests.

## Authors' contributions

EM carried out the implementation of the idea, contributed in the development of new characteristic index for discriminating different pathology of signals, collected data from different standard databases for analysis, and drafted the manuscript. SY participated in the design of the study and interpretation of data, and was involved in critically revising the manuscript. MK conceived of the study, and participated in its design, analysis and interpretation of data, defined mathematical index to be used for discriminating arrhythmias, and helped to draft and finalize the manuscript. All authors read and approved the final manuscript.

## Appendix

The steps involved in the EMD technique are described below using an example.

**Figure 11:**
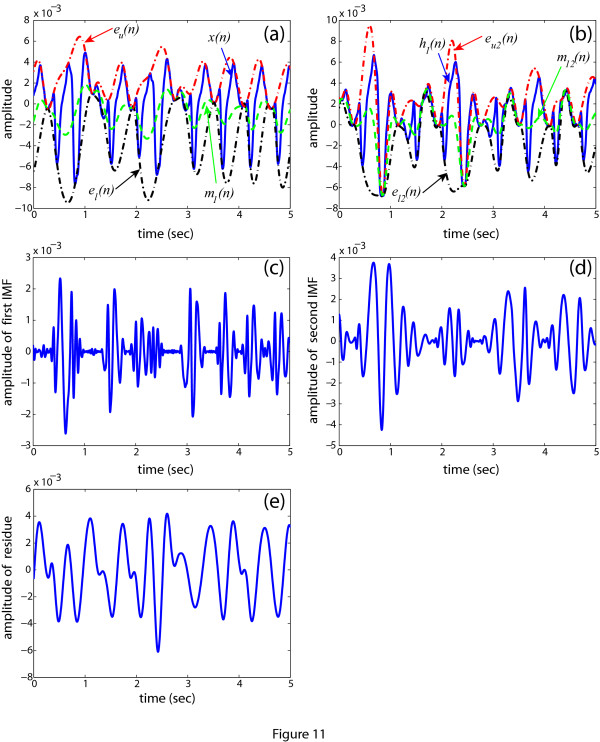
**Illustration of the EMD using an example**. (a) The original signal *x*(*n*), two envelopes *e_u_*(*n*) and *e_l_*(*n*), and the mean of the envelope *m*_1_(*n*); (b) The first component *h*_1_(*n*), two envelopes *e*_*u*2_(*n*) and *e*_*l2*_(*n*), and the mean envelope *m*_12_(*n*); (c) The first IMF *imf*_1_(*n*); (d) The second IMF *imf*_2_(*n*); (e) The residue.

1. Determine the upper envelop *e_u_*(*n*) and the lower envelop *e_l_*(*n*). These two envelopes are shown in Figure 11(a) along with the original signal *x*(*n*).

2. Determine the mean of the envelope, i.e., *m*_1_(*n*) = [*e_u_*(*n*) + *e_l_*(*n*)]/2. The variation of *m*_1_(*n*) is displayed in Figure 11(a).

3. Extract the first component *h*_1_(*n*) using eqn. (3).

4. Ideally, *h*_1_(*n*) should be the first IMF. But, it is observed from Figure 11(b) that the *h*_1_(*n*) does not satisfy the conditions of an IMF.

5. Now, treat *h*_1_(*n*) as *x*(*n*) in step (1). Determine the two envelopes from *h*_1_(*n*) and the mean of these two envelopes (Figure 11(b)). After subtraction of the mean from the *h*_1_(*n*) a new signal *h*_1(2)_(*n*) is obtained. Now, check the conditions of an IMF and also calculate the value of SD from eqn. (4), where *h*_1(1)_=*h*_1_(*n*).

6. Continue the process until *h*_1(*k*-1) _satisfies the conditions of the IMFs. When the conditions are satisfied, the first IMF is found as shown in Figure 11(c). Now, the first IMF is subtracted from the initial signal *x*(*n*).

7. The second IMF (Figure 11(d)) is extracted following the steps (1) to (6) except that the subtracted signal is used instead of *x*(*n*). No further decomposition is performed here as we need two IMFs for our analysis. The residue of the EMD is shown in Figure 11(e).
